# A time-delayed epidemic model for control of highly pathogenic avian influenza (HPAI H5Nx) with vaccination, compliance, and non-pharmaceutical interventions

**DOI:** 10.3389/fpubh.2026.1819528

**Published:** 2026-06-22

**Authors:** Oluwatosin Babasola, Mohamed Bakheet, Christina Næsborg-Nielsen, Sachin Subedi, M H M Mubassir, Justin Bahl

**Affiliations:** 1 Department of Infectious Diseases, College of Veterinary Medicine, University of Georgia, Athens, GA, United States; 2 Center for Ecology of Infectious Diseases, University of Georgia, Athens, GA, United States; 3 Department of Epidemiology and Biostatistics, University of Georgia, Athens, GA, United States; 4 Institute of Bioinformatics, University of Georgia, Athens, GA, United States

**Keywords:** compliance, epidemic preparedness, highly pathogenic avian influenza, human infection, non-pharmaceutical interventions, time-delayed model, vaccination

## Abstract

**Introduction:**

Highly pathogenic avian influenza poses an ongoing zoonotic risk with the potential for severe human outbreaks. Existing epidemic models often assume immediate vaccine effectiveness and homogeneous populations, which may overestimate the impact of vaccination during the early phase of an outbreak. This study develops a time-delayed epidemic model to examine how vaccination timing, delayed immune protection, behavioral heterogeneity, and non-pharmaceutical interventions jointly shape the transmission dynamics of a hypothetical human outbreak of highly pathogenic avian influenza H5Nx.

**Methods:**

A time-delayed epidemic model was developed that incorporates incubation delay, vaccine deployment delay, and vaccine-to-protection delay. The population was stratified into vaccine-accepting and vaccine-hesitant groups to represent behavioral heterogeneity in vaccine uptake. The model was analyzed through reproduction number and stability analyses, and numerical simulations were performed to evaluate the effects of delays on peak infectious burden, cumulative infections, epidemic timing, and intervention effectiveness.

**Results:**

The results showed that delays in vaccine deployment and immune protection substantially reduced the effectiveness of vaccination in limiting peak infection burden. Earlier vaccine deployment reduced transmission during the initial growth phase, whereas delayed deployment shifted the impact of vaccination to later stages of the epidemic. Higher vaccination acceptance reduced both peak burden and cumulative infections, while vaccine-hesitant individuals consistently experienced higher attack rates. Non-pharmaceutical interventions reduced transmission immediately and exerted the strongest influence during the early phase of the epidemic. The incubation delay altered epidemic timing and transient dynamics without changing the invasion threshold.

**Discussion:**

The findings demonstrate that epidemic outcomes depend strongly on intervention timing, the rate at which immune protection develops, and population behavior. Delays associated with vaccine availability and immune response can substantially diminish the short-term benefits of vaccination, particularly when outbreak growth is rapid. Incorporating behavioral heterogeneity and multiple delay mechanisms provides a more realistic basis for evaluating intervention strategies and strengthening preparedness for future human outbreaks of highly pathogenic avian influenza.

## 1 Introduction

Highly pathogenic avian influenza A H5Nx virus remains a persistent global concern due to sustained transmission among wild birds, domestic poultry, and mammals, with sporadic zoonotic infections in humans. Although no human to human transmission has been recorded, the increasing geographic spread and frequency of outbreaks indicate continued viral evolution with uncertain public health consequences (1, 2). Foundational studies of influenza ecology and evolution identify that cross-species transmission and viral adaptation as central mechanisms shaping pandemic risk (3, 4), introducing substantial uncertainty in the timing, scales, and severity of potential outbreaks (5–7).

Recent pandemics such as the 2009 H1N1 and COVID-19 demonstrate that vaccine development and deployment of vaccines for emerging pathogens are subject to substantial delays (8, 9). During the 2009 H1N1 pandemic, significant lags occurred between strain identification, vaccine production, and large scale rollout (10). Similar patterns were observed during the COVID-19 pandemic, where early population-level impact was constrained by manufacturing capacity, distribution limitations and the time required to achieve protective immunity (11). Although clinical trials reported high individual-level efficacy for mRNA vaccines (12), reductions in transmission emerged gradually as vaccination coverage increased (13). These observations highlight that intervention timing plays a critical role in shaping epidemic trajectories.

Vaccination remains a central tool for mitigating transmission and disease burden during outbreaks in both human and animal populations (14, 15). In particular, regions such as China, Egypt, Indonesia, and Vietnam have adopted vaccination as a key strategy for controlling avian influenza in poultry populations (15). More recently, Finland has extended avian influenza vaccination to humans in high-risk occupational groups, including fur and poultry farm workers (16). However, vaccination is not yet widely adopted globally, and its effectiveness depends not only on coverage but also on the speed at which protection is achieved (17, 18). Also, (19) demonstrates that vaccination can reduce transmission intensity and decrease epidemic peaks when sufficient coverage is established. For emerging zoonotic infections such as HPAI, vaccines are typically unavailable during the early stages of human transmission (20). As a result, delays associated with antigen selection, production, distribution, and immune response development can therefore reduce population-level effectiveness (21). Several modeling studies further suggest that delayed vaccine rollout is associated with larger epidemic size and higher peak burden (22, 23) which indicate that models assuming immediate vaccine-induced protection may overestimate control effectiveness.

Beyond vaccination timing, outbreak dynamics are shaped by interactions between intervention processes and the natural progression of infection (24, 25). These interactions are inherently non-linear, limiting the ability to infer epidemic dynamics from isolated interventions (26). Consequently, quantitative models are required to evaluate how intervention timing and adherence jointly influence outbreak size, peak timing, and control feasibility. As such, compartmental models provide a useful framework by linking mechanistic assumptions to observable epidemiological outcomes (27). While the extensions of these models have incorporated vaccination, waning immunity, and behavioral responses, many still rely on the simplifying assumption that transitions occur instantaneously. For infections with latent periods and delayed immune protection, this assumption can distort epidemic dynamics and bias estimates of control thresholds (28–30). Time-delay models provide a natural extension for this assumption by explicitly representing the biological processes. Existing studies have shown that delays can alter stability properties, epidemic thresholds, and long term dynamics of the system (31, 32). In particular, delayed immune protection following vaccination increases the level of coverage required to achieve epidemic control (33). However, most delay-based models focus primarily on biological processes and rarely incorporate delays associated with vaccine deployment and campaign initiation.

In addition to structural model assumptions, behavioral factors could also influence intervention effectiveness by shaping vaccination uptake and adherence to non-pharmaceutical interventions. Variability in these behaviors can substantially reduce the effectiveness of control measures (34, 35). This effect is particularly pronounced during the early phase of an outbreak, when non-pharmaceutical interventions such as hygiene practices and contact reduction are often implemented before vaccines become available (36). Although these measures can reduce early transmission, their impact depends on sustained adherence and coordination with subsequent vaccination strategies (37). Consequently, epidemic dynamics depend not only on intervention timing but also on the interaction between behavioral responses and delayed vaccination.

Despite the recognized importance of these factors, existing mathematical models of avian influenza do not fully capture their combined effects. Most models focus on between hosts transmission, with limited attention to human epidemic dynamics under delayed vaccination (38, 39). Even in models that consider human transmission, vaccination processes are often simplified or treated without explicit delays (40–42). As a result, the joint effects of multiple delays, vaccination compliance, and non-pharmaceutical interventions remain insufficiently represented, limiting the ability of current models to evaluate how these mechanisms interact to shape epidemic control. To address this gap, there is a need for modeling approaches that integrate delayed vaccination, behavioral heterogeneity, and non-pharmaceutical interventions within a unified framework. Such an approach is essential given the potential for future HPAI strains to acquire efficient human to human transmission (43), where intervention timing and compliance could directly influence outbreak dynamics.

In response to this need, this study develops a time-delayed epidemic model to examine the effects of delayed vaccination on HPAI transmission in humans. The model distinguishes individuals who are vaccinated but not yet protected, which enables explicit representation of the vaccine-to-protection delay. It also incorporates incubation delay and pre-deployment delay associated with vaccine availability and rollout. Vaccination compliance and non-pharmaceutical interventions are further included to represent behavioral and policy influences on transmission. Using this framework, we quantify how these factors affect outbreak size, peak timing, and conditions for epidemic control. We further evaluates the extent to which non-pharmaceutical interventions can mitigate the impact of delayed vaccination.

The remainder of the paper is organized as follows. Section 2 presents the methods and discuss the model formulation with the analytical framework. Section 3 presents the numerical simulations and quantitative findings while section 4 gives the discussion which interprets the results in relation to existing literature and public health implications. Section 5 provides the conclusion, and Section 6 outlines directions for future work.

## 2 Methods

Mathematical models are widely used to describe transmission dynamics and to evaluate intervention strategies in infectious disease systems. In many studies, epidemic dynamics are represented using ordinary differential equation models, which assume that transitions between disease states occur instantaneously. This assumption simplifies analysis but does not always reflect key biological and operational processes associated with influenza transmission. In particular, infection progression involves a latent incubation period before individuals become infectious, and vaccination does not confer immediate protection due to the time required for immune response development. In addition, delays in vaccine availability arising from production and distribution introduce time lags that cannot be captured using instantaneous transition models (31–33).

These limitations indicate the need for modeling approaches that can explicitly represent time dependent processes in disease transmission and control. Delay differential equations provide an extension of classical epidemic models by allowing system dynamics to depend on past states. Through this representation, incubation periods, delayed immune protection, and delayed intervention implementation can be incorporated directly into the model structure. As a result, delay-based models provide a more biologically consistent description of epidemic progression and intervention timing.

Building on this approach, this section develops a time-delayed model that incorporates three distinct delays, namely the incubation delay, the vaccine-to-protection delay, and the pre-deployment delay associated with vaccine availability. In addition to these temporal features, the model includes vaccination compliance and non-pharmaceutical interventions to capture behavioral and policy influences on transmission. This formulation is then used to examine how the interaction between biological delays, intervention timing, and behavioral responses influences outbreak dynamics, infection burden, and peak timing.

### 2.1 Model description

We consider a time-delayed model that describe the transmission dynamics of an HPAI outbreak within a human population. We define the total population at time *t* as *N*(*t*), and this is divided into two behavioral groups based on vaccine decision making, namely vaccine-accepting and vaccine-hesitant individuals. Then, we have the total population in Equation 1 as the sum of all compartments which is given as


$$\begin{array}{l} N\left(t\right)=S\left(t\right)+W\left(t\right)+P\left(t\right)+E\left(t\right)+I\left(t\right)+R\left(t\right), \end{array}$$ (1)

where *S*(*t*) = *S*_*a*_(*t*)+*S*_*h*_(*t*), *W*(*t*) = *W*_*a*_(*t*), *P*(*t*) = *P*_*a*_(*t*), *E*(*t*) = *E*_*a*_(*t*)+*E*_*h*_(*t*), *I*(*t*) = *I*_*a*_(*t*)+*I*_*h*_(*t*), and *R*(*t*) = *R*_*a*_(*t*)+*R*_*h*_(*t*). The subscripts *a* and *h* denote the vaccine-accepting and vaccine-hesitant groups respectively. This structure separates infection dynamics from vaccination related processes. The susceptible class *S*(*t*) contains individuals at risk of infection. In the accepting group, susceptible individuals receive vaccine and enter the waiting class *W*(*t*). This class represents vaccinated individuals who have not yet developed protective immunity. After this stage, individuals move to the protected class *P*(*t*), where susceptibility to infection is reduced but not eliminated. The exposed class *E*(*t*) consists of infected individuals who are not yet infectious, while the infectious class *I*(*t*) consists of individuals capable of transmitting infection. Finally, the recovered class *R*(*t*) consists of individuals who are assumed to remain immune over the time horizon considered.

This formulation allows the delayed effect of vaccination to be represented explicitly. Individuals in the waiting class remain susceptible until protective immunity becomes active, whereas individuals in the protected class may still experience infection but at a reduced rate. In contrast, vaccine-hesitant individuals do not pass through vaccination stages and follow the infection process without vaccination. This distinction allows the model to isolate the effects of delayed protection and incomplete vaccine uptake on transmission dynamics.

### 2.2 Incorporation of time delays

Building on the compartmental structure previously described, the model incorporates three delays that represent key biological and operational processes. These delays reflect the fact that infection progression and intervention effects occur over finite time intervals rather than instantaneously, and therefore they are represented explicitly to capture realistic epidemic dynamics.

The first delay denoted by τ_*E*_, represents the incubation period between infection and the onset of infectiousness. During this period, individuals remain in the exposed class and do not contribute to transmission, so this delay determines when newly infected individuals begin to influence epidemic growth. The second delay denoted by τ_*V*_, represents the vaccine-to-protection delay. After vaccination, individuals enter the waiting class and require a finite period before protective immunity becomes effective. This implies that they remain fully susceptible throughout this interval. As a result, only uninfected individuals move to the protected class, where susceptibility is reduced but not eliminated. Finally, we have the pre-deployment delay denoted by τ_*P*_ which is associated with vaccine rollout. This delay captures the time between the onset of the epidemic and the implementation of vaccination, which is constraints by vaccine development, approval, and distribution. As such, vaccination begins only after this delay has elapsed.

It is important to note that these delays are operated at different stages of the epidemic and influence transmission dynamics through distinct mechanisms. The incubation delay determines the timing of infectiousness and therefore affects the rate at which new infections contribute to transmission. The pre-deployment delay governs when vaccination is introduced, and the vaccine-to-protection delay determines how quickly vaccinated individuals acquire effective immunity. In general, the time-delays determine how intervention timing interacts with infection dynamics to shape outbreak size and peak timing.

### 2.3 Vaccination and non-pharmaceutical interventions

Vaccination and non-pharmaceutical interventions are further incorporated as control mechanisms that act on susceptible individuals with imperfect uptake. This is represented through the vaccination compliance parameter *c*_*v*_∈[0, 1], which denotes the proportion of susceptible individuals who are willing and able to be vaccinated once vaccine becomes available. A value of *c*_*v*_ = 0 corresponds to no uptake, whereas *c*_*v*_ = 1 corresponds to full compliance. This formulation is consistent with population-level models of behavioral adherence to interventions (34, 35). Given this definition, the per capita vaccination rate among compliant individuals is denoted by ν, which implies that the effective vaccination rate is *c*_*v*_ν.

Following vaccination, individuals enter the waiting class and remain there until protective immunity becomes active. During this period, vaccinated individuals remain fully susceptible, reflecting the delay between vaccine administration and immune response. After this period, individuals transition to the protected class, where vaccine-induced protection is assumed to be partial. This partial protection is represented by a residual susceptibility parameter ϵ∈[0, 1], where ϵ = 0 corresponds to complete protection and larger values indicate reduced vaccine effectiveness.

Similarly, non-pharmaceutical interventions are incorporated as population-level measures that reduce effective transmission. These measures capture the combined effects of behavioral and policy driven actions. This is represented by a compliance parameter *c*_*n*_ ∈ [0, 1], which denotes the proportion of the population adhering to these interventions. Consequently, the transmission rate is scaled by a factor (1−*c*_*n*_), so that higher compliance leads to lower effective transmission. This combined representation of vaccination and non-pharmaceutical interventions enables the model to capture both delayed protection and immediate transmission reduction within a unified structure. With these mechanisms defined, we then presents the full mathematical formulation of the model.

### 2.4 Model formulation

**Figure 1:**
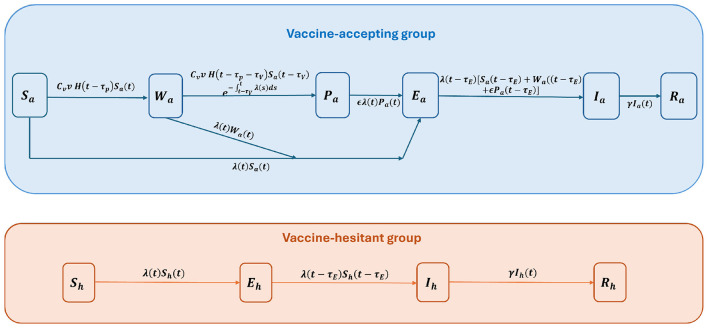
Schematic representation of the time-delayed HPAI model corresponding to the system in Equation 3. The subscripts *a* and *h* denote the vaccine accepting and vaccine hesitant groups, respectively. The diagram shows the susceptible, vaccinated waiting, protected, exposed, infectious, and recovered pathways, together with the delayed vaccination and incubation transitions.

We consider a closed population structured into two behavioral groups based on vaccine decisions. The vaccine-accepting group and the vaccine-hesitant group, where both groups are considered to follow similar infection process. The transmission is governed by the force of infection λ(*t*), which is defined as


$$\begin{array}{l} \lambda \left(t\right)=\beta \left(1-c_{n}\right)\frac{I\left(t\right)}{N\left(t\right)}, \end{array}$$ (2)

where β represent the transmission rate and *c*_*n*_ represents compliance with non-pharmaceutical interventions. The model is formulated such that the vaccination is introduced after the pre-deployment delay τ_*P*_, which is represented with a Heaviside function *H*(*t*−τ_*P*_). Following this initiation, susceptible individuals in the vaccine-accepting group are vaccinated at rate *c*_*v*_ν and enter the waiting class. Individuals in this class remain susceptible until protective immunity becomes effective after a delay τ_*V*_. The resulting system of delay differential equations is given in Equation 3 while the schematic representation of the compartmental transition structure is shown in Figure 1


$$\begin{array}{l} \frac{dS_{a}}{dt}=-\lambda \left(t\right)S_{a}\left(t\right)-c_{v}\nu H\left(t-\tau_{P}\right)S_{a}\left(t\right),\\ \frac{dW_{a}}{dt}=c_{v}\nu H\left(t-\tau_{P}\right)S_{a}\left(t\right)-\lambda \left(t\right)W_{a}\left(t\right)\\ \;-c_{v}\nu H\left(t-\tau_{P}-\tau_{V}\right)S_{a}\left(t-\tau_{V}\right)\exp\left(-\int_{t-\tau_{V}}^{t}\lambda \left(s\right)ds\right),\\ \frac{dP_{a}}{dt}=c_{v}\nu H\left(t-\tau_{P}-\tau_{V}\right)S_{a}\left(t-\tau_{V}\right)\exp\left(-\int_{t-\tau_{V}}^{t}\lambda \left(s\right)ds\right)\\ \;-\epsilon \lambda \left(t\right)P_{a}\left(t\right),\\ \frac{dE_{a}}{dt}=\lambda \left(t\right)(S_{a}\left(t\right)+W_{a}\left(t\right)+\epsilon P_{a}\left(t\right))-\lambda \left(t-\tau_{E}\right)(S_{a}\left(t-\tau_{E}\right)\\ \;+W_{a}\left(t-\tau_{E}\right)+\epsilon P_{a}\left(t-\tau_{E}\right)),\\ \frac{dI_{a}}{dt}=\lambda \left(t-\tau_{E}\right)(S_{a}\left(t-\tau_{E}\right)+W_{a}\left(t-\tau_{E}\right)+\epsilon P_{a}\left(t-\tau_{E}\right))\\ \;-\gamma I_{a}\left(t\right),\\ \frac{dR_{a}}{dt}=\gamma I_{a}\left(t\right),\\ \frac{dS_{h}}{dt}=-\lambda \left(t\right)S_{h}\left(t\right),\\ \frac{dE_{h}}{dt}=\lambda \left(t\right)S_{h}\left(t\right)-\lambda \left(t-\tau_{E}\right)S_{h}\left(t-\tau_{E}\right),\\ \frac{dI_{h}}{dt}=\lambda \left(t-\tau_{E}\right)S_{h}\left(t-\tau_{E}\right)-\gamma I_{h}\left(t\right),\\ \frac{dR_{h}}{dt}=\gamma I_{h}\left(t\right). \end{array}$$ (3)

The exponential term


$$\exp\left(-\int_{t-\tau_{V}}^{t}\lambda \left(s\right)ds\right)$$

represents the probability that a vaccinated individual remains uninfected throughout the vaccine-to-protection interval. Since λ(*s*) defined in Equation 2 denotes the instantaneous force of infection at time *s*, the integral gives the cumulative infection hazard experienced during the waiting period.

Under this assumption, demographic processes such as births and natural deaths are neglected, since the model focuses on transmission dynamics over a 1-year epidemic horizon. During this period, HPAI outbreak dynamics are primarily governed by infection, recovery, and intervention processes rather than demographic turnover (44). As a result, this time horizon would be sufficient to capture the outbreak phase, peak timing, and cumulative infection burden associated with a single epidemic wave. Reinfection is also not included, since post-infection immunity is assumed to persist over the duration of the outbreak and the probability of homologous reinfection is negligible (45). Although influenza viruses evolve over time, antigenic drift and strain replacement typically occur across multiple epidemic seasons rather than within a single wave (46, 47). These assumptions ensure that the model isolates the effects of delayed vaccination and behavioral heterogeneity on outbreak dynamics without additional confounding processes.

In the numerical illustrations, the epidemic is simulated over a 1-year horizon. This time scale is short relative to demographic processes such as births and natural deaths, but long enough to capture the outbreak phase, peak timing, and cumulative infection burden considered in this study.

With the full model specified, the next subsection examines its analytical properties, such as well-posedness, biological feasibility, and threshold dynamics that characterize the conditions for disease persistence or elimination.

### 2.5 Model analysis

This subsection establishes that the model system in Equation 3 is well-defined and its solutions remain biologically meaningful over time. To formalize this, we begin by defining the state variables and delay structure of the system. Suppose the state vector be given by


$$\begin{array}{l} x\left(t\right)=(S_{a}\left(t\right),W_{a}\left(t\right),P_{a}\left(t\right),E_{a}\left(t\right),I_{a}\left(t\right),R_{a}\left(t\right),S_{h}\left(t\right),E_{h}\left(t\right),I_{h}\left(t\right),\\ R_{h}\left(t\right){)}^{⊤}, \end{array}$$

which comprises of all compartments for both behavioral groups. The set of delay parameters is defined as


$$T=\left\{\tau_{E},\tau_{V},\tau_{P}\right\},$$

and the maximal delay governing the system is given by


$$\tau =\max\left\{\tau_{E},\tau_{V},\tau_{P}+\tau_{V}\right\}.$$

Given this delay structure, the system requires specification of an initial history over the interval [−τ, 0]. Accordingly, we assume that the initial condition is provided by a continuous function defined as


x(θ)=ϕ(θ) for all θ∈[-τ,0],

where $\phi \left(\theta \right)\in {\mathbb{R}}_{\geq 0}^{10}$ and


$${\mathbb{R}}_{\geq 0}^{10}=\left\{x\in {\mathbb{R}}^{10}:x_{i}\geq 0\text{ for }i=1,\ldots ,10\right\}.$$

This ensures that all state variables are non-negative at the initial time and remain consistent. we then establish the existence, uniqueness, and biological feasibility of solutions to the system in Equation 3.

#### 2.5.1 Well-posedness and biological feasibility

Here, we establish that the system in Equation 3 is well-posed and that its solutions remain biologically feasible for all future time.

**Theorem 1** (well-posedness and biological feasibility). *Suppose that all parameters are non-negative, with {*c*_*n*_, *c*_*v*_} ∈ [0, 1], and that the initial history ϕ(θ) is continuous on [−τ, 0], componentwise non-negative, and biologically admissible. Then the system in Equation 3 admits a unique global solution*


$$x\left(t\right)\in C\left(\left[-\tau ,\infty \right),{\mathbb{R}}^{10}\right),$$

*which remains non-negative for all *t* ≥ 0. Moreover, the total population satisfies*


N(t)=N(0) for all t≥0,

*and the feasible region*


$$\Omega =\left\{x\in {\mathbb{R}}_{\geq 0}^{10}:\overset{10}{\underset{k=1}{\sum }}x_{k}=N\left(0\right)\right\}$$

*is positively invariant*.

*Proof*. Since the system in Equation 3 contains delayed terms that depend on past states, ordinary differential equation theory is not sufficient for establishing well-posedness. We therefore reformulate the system as a retarded functional differential equation, where the current rate of change depends on the recent history of the solution. This formulation allows standard existence and uniqueness theory for delay differential equations to be applied. Accordingly, we rewrite the system in Equation 3 as a retarded functional differential equation of the form


$$\frac{dx\left(t\right)}{dt}=F\left(t,x_{t}\right),$$

where $x_{t}\in C\left(\left[-\tau ,0\right],{\mathbb{R}}^{10}\right)$ is defined by *x*_*t*_(θ) = *x*(*t*+θ) for θ ∈ [−τ, 0]. Then, the proof is divided into four steps. First, we establish local existence and uniqueness. Next, we show non-negativity of solutions. We then prove boundedness and positive invariance of the feasible region. Finally, we deduce global existence.

*1. Existence and uniqueness*

For each fixed *t* ≥ 0, the functional *F*(*t*, ·) depends on *x*_*t*_ through evaluation at finitely many delay points and the exponential survival term


$$\exp\left(-\int_{t-\tau_{V}}^{t}\lambda \left(s\right)ds\right),$$

where


$$\lambda \left(t\right)=\beta \left(1-c_{n}\right)\frac{I\left(t\right)}{N\left(t\right)}$$

is continuous whenever the state variables are continuous and *N*(*t*)>0. Since evaluation maps on *C*([−τ, 0], ℝ^10^) are continuous linear operators, and sums, products, and compositions of locally Lipschitz functions remain locally Lipschitz, it follows that *F*(*t*, ·) is locally Lipschitz on bounded subsets of *C*([−τ, 0], ℝ^10^). The explicit dependence on *t* enters through the Heaviside functions *H*(*t*−τ_*P*_) and *H*(*t*−τ_*P*_−τ_*V*_), which are bounded and measurable. Therefore, *F* satisfies the Carathéodory conditions for retarded functional differential equations. By standard existence and uniqueness results for finite delay systems (48, 49), there exists a unique maximal solution


$$x\left(t\right)\in C\left(\left[-\tau ,t_{\max}\right),{\mathbb{R}}^{10}\right)$$

for some *t*_max_>0.

*2. Non-negativity of solutions*

We now show that each component remains non-negative on [0, *t*_max_).

For the susceptible classes, direct integration gives


$$S_{a}\left(t\right)=S_{a}\left(0\right)\exp\left(-\int_{0}^{t}\left[\lambda \left(s\right)+c_{v}\nu H\left(s-\tau_{P}\right)\right]ds\right)\geq 0,$$

and


$$S_{h}\left(t\right)=S_{h}\left(0\right)\exp\left(-\int_{0}^{t}\lambda \left(s\right)ds\right)\geq 0.$$

For the protected class, we define


$$q\left(s\right)=c_{v}\nu H\left(s-\tau_{P}-\tau_{V}\right)S_{a}\left(s-\tau_{V}\right)\exp\left(-\int_{s-\tau_{V}}^{s}\lambda \left(\xi \right)d\xi \right).$$

The quantity *q*(*s*) represents the inflow into the protected class at time *s*, namely the vaccinated individuals who entered the waiting class at time *s*−τ_*V*_ and remained uninfected throughout the interval [*s*−τ_*V*_, *s*]. Since all factors are non-negative, we have *q*(*s*) ≥ 0. Applying variation of constants to the equation for *P*_*a*_, we obtain


$$\begin{array}{l} P_{a}\left(t\right)=P_{a}\left(0\right)\exp\left(-\int_{0}^{t}\epsilon \lambda \left(s\right)ds\right)+\int_{0}^{t}q\left(s\right)\exp\left(-\int_{s}^{t}\epsilon \lambda \left(\xi \right)d\xi \right)\\ \;ds\geq 0. \end{array}$$

For the infectious classes, we define


$$F_{a}\left(r\right)=\lambda \left(r\right)\left(S_{a}\left(r\right)+W_{a}\left(r\right)+\epsilon P_{a}\left(r\right)\right),\;F_{h}\left(r\right)=\lambda \left(r\right)S_{h}\left(r\right).$$

Here, *F*_*a*_(*r*) and *F*_*h*_(*r*) represent the rates at which new infections are generated at time *r* in the vaccine-accepting and vaccine-hesitant groups, respectively. Hence


$$F_{a}\left(s-\tau_{E}\right)=\lambda \left(s-\tau_{E}\right)\left(S_{a}\left(s-\tau_{E}\right)+W_{a}\left(s-\tau_{E}\right)+\epsilon P_{a}\left(s-\tau_{E}\right)\right),$$

$$F_{h}\left(s-\tau_{E}\right)=\lambda \left(s-\tau_{E}\right)S_{h}\left(s-\tau_{E}\right),$$

which are precisely the delayed inflow terms into the infectious classes. Using variation of constants,


$$I_{a}\left(t\right)=I_{a}\left(0\right)e^{-\gamma t}+\int_{0}^{t}e^{-\gamma \left(t-s\right)}F_{a}\left(s-\tau_{E}\right)ds\geq 0,$$

and


$$I_{h}\left(t\right)=I_{h}\left(0\right)e^{-\gamma t}+\int_{0}^{t}e^{-\gamma \left(t-s\right)}F_{h}\left(s-\tau_{E}\right)ds\geq 0.$$

For the recovered classes


$$\begin{array}{l} R_{a}\left(t\right)=R_{a}\left(0\right)+\int_{0}^{t}\gamma I_{a}\left(s\right)ds\geq 0,\\ R_{h}\left(t\right)=R_{h}\left(0\right)+\int_{0}^{t}\gamma I_{h}\left(s\right)ds\geq 0. \end{array}$$

For the exposed classes, using the identities


$$\frac{dE_{a}}{dt}=F_{a}\left(t\right)-F_{a}\left(t-\tau_{E}\right),\;\frac{dE_{h}}{dt}=F_{h}\left(t\right)-F_{h}\left(t-\tau_{E}\right),$$

and the biological consistency of the initial history, we represent the delayed classes as


$$E_{a}\left(t\right)=\int_{t-\tau_{E}}^{t}F_{a}\left(s\right)ds\geq 0,\;E_{h}\left(t\right)=\int_{t-\tau_{E}}^{t}F_{h}\left(s\right)ds\geq 0.$$

These representations show that *E*_*a*_(*t*) and *E*_*h*_(*t*) measure the total number of individuals infected during the previous incubation interval who have not yet progressed to infectiousness.

Finally, for the waiting class, we define


$$u\left(s\right)=c_{v}\nu H\left(s-\tau_{P}\right)S_{a}\left(s\right),$$

which represents the inflow of newly vaccinated susceptible individuals into the waiting class at time *s*. Using the equation for *W*_*a*_ together with the consistency of the initial history, we obtain


$$W_{a}\left(t\right)=\int_{t-\tau_{V}}^{t}u\left(s\right)\exp\left(-\int_{s}^{t}\lambda \left(\xi \right)d\xi \right)ds\geq 0.$$

This representation shows that *W*_*a*_(*t*) consists of individuals vaccinated during the interval [*t*−τ_*V*_, *t*] who remain uninfected and have not yet completed the vaccine-to-protection delay. Therefore, all components of *x*(*t*) remain non-negative for all *t* ∈ [0, *t*_max_).

*3. Boundedness and positive invariance*

Let


$$N\left(t\right)=\overset{10}{\underset{k=1}{\sum }}x_{k}\left(t\right).$$

By summing all equations in Equation 3, every transfer term cancels exactly, yielding


$$\frac{dN\left(t\right)}{dt}=0.$$

Hence


N(t)=N(0) for all t≥0.

Since each component is non-negative and their sum remains constant, it follows that


$$0\leq x_{k}\left(t\right)\leq N\left(0\right),\;k=1,\ldots ,10.$$

Therefore,


$$\Omega =\left\{x\in {\mathbb{R}}_{\geq 0}^{10}:\overset{10}{\underset{k=1}{\sum }}x_{k}=N\left(0\right)\right\}$$

is positively invariant.

*4. Global existence*

Because the solution remains bounded in the positively invariant set Ω, no component can blow up in finite time. Since the vector field is locally Lipschitz on bounded sets, the standard continuation theorem for delay differential equations (48), implies that the maximal existence time satisfies *t*_max_ = ∞. Therefore, the system in Equation 3 admits a unique global solution


$$x\left(t\right)\in C\left(\left[-\tau ,\infty \right),{\mathbb{R}}^{10}\right),$$

which remains non-negative and bounded for all *t* ≥ 0, and the feasible region Ω is positively invariant.

Having established that the model is well-posed and biologically feasible, we next examine the local stability properties of the disease free set and derive the threshold quantities that govern disease invasion and persistence.

### 2.6 Stability analysis

Here, we analyze the behavior of the system in Equation 3 in a neighborhood of the disease free set and derive the threshold quantities that determine whether infection can invade the population.

This analysis determines whether a small number of infected individuals introduced into a largely susceptible population will die out or generate epidemic growth. The resulting threshold quantities therefore provide a mathematical characterization of invasion and control conditions. This is performed on the positively invariant feasible region Ω established in Theorem 1.

#### 2.6.1 Disease free set and linearized infected subsystem

We begin by characterizing the disease free set, which consists of all states in Ω for which no exposed or infectious individuals are present. Thus,


$$D=\left\{x\in \Omega :E_{a}=I_{a}=E_{h}=I_{h}=0\right\}.$$

Let


$$x^{0}=\left(S_{a}^{0},W_{a}^{0},P_{a}^{0},0,0,R_{a}^{0},S_{h}^{0},0,0,R_{h}^{0}\right)\in D,$$

where


$$S_{a}^{0}+W_{a}^{0}+P_{a}^{0}+R_{a}^{0}+S_{h}^{0}+R_{h}^{0}=N_{0}:=N\left(0\right)>0.$$

This state represents an arbitrary disease free configuration of the population, with the distribution across susceptible, vaccinated, protected, and recovered classes determined by the initial conditions and vaccination history. To study whether infection can invade near this disease free state, we consider on the infected compartments during the initial phase of transmission. Near this state, infected variables remain small, whereas the uninfected compartments vary only through higher order effects. Therefore, the initial growth (decay) of infection is governed by the linearized equations for the infected compartments. Consequently, the local invasion dynamics are characterized by the linearized infected subsystem obtained from the total infectious population.


$$I\left(t\right)=I_{a}\left(t\right)+I_{h}\left(t\right).$$

Then, the system in Equation 3 gives


$$\begin{array}{l} \frac{dI}{dt}=\lambda (t-\tau_{E})(S_{a}(t-\tau_{E})+W_{a}(t-\tau_{E})+\epsilon P_{a}(t-\tau_{E})\\ \;+\;S_{h}(t-\tau_{E}))-\gamma I(t). \end{array}$$ (4)

The Equation 4 describes the rate of change of the total infectious population as the balance between delayed inflow from newly infectious individuals and removal through recovery.

We now linearize this expression about the disease free state *x*^0^. Near D, the infected compartments are small, and the uninfected compartments differ from their disease free values only by higher order terms. Therefore, to first order, we approximate


$$S_{a}\left(t-\tau_{E}\right)+W_{a}\left(t-\tau_{E}\right)+\epsilon P_{a}\left(t-\tau_{E}\right)+S_{h}\left(t-\tau_{E}\right)\approx S_{a}^{0}+W_{a}^{0}+\epsilon P_{a}^{0}+S_{h}^{0}.$$

We then define


$$S_{\text{eff}}^{0}=S_{a}^{0}+W_{a}^{0}+\epsilon P_{a}^{0}+S_{h}^{0},$$

which represents the effective susceptible population available for transmission at the disease free state.

Since *N*(*t*) = *N*_0_ throughout the feasible region Ω, the force of infection satisfies the first order approximation


$$\lambda \left(t-\tau_{E}\right)\approx \beta \left(1-c_{n}\right)\frac{I\left(t-\tau_{E}\right)}{N_{0}}.$$

Substituting these approximations into the equation for *I*(*t*) yields the linear delay equation


$$\begin{array}{l} \frac{dI}{dt}=\beta \left(1-c_{n}\right)\frac{S_{\text{eff}}^{0}}{N_{0}}I\left(t-\tau_{E}\right)-\gamma I\left(t\right). \end{array}$$ (5)

This governs the initial growth (decay) of infection near the disease free set. This linearization retains only first order terms in the infected variables and therefore identifies the components that directly determine invasion. In particular, τ_*E*_ remains in the reduced infected subsystem because it governs the progression from infection to infectiousness. By contrast, the delays τ_*P*_ and τ_*V*_ do not appear explicitly in Equation 5, since they influence the linearized dynamics only indirectly through the disease free quantities $W_{a}^{0}$ and $P_{a}^{0}$ that contribute to $S_{\text{eff}}^{0}$.

#### 2.6.2 Basic and effective reproduction numbers

The linear delay in Equation 5 previously derived characterizes the initial growth (decay) of infection near the disease free set. A standard approach to assessing whether infection can invade is to define threshold quantities that compare the rate of infection generation with the rate of removal. These thresholds are expressed in terms of reproduction numbers, which quantify the expected number of secondary infections produced by a typical infectious individual under specified conditions.

So, we first define the controlled basic reproduction number by


$$\begin{array}{l} R_{0}=\frac{\beta \left(1-c_{n}\right)}{\gamma }. \end{array}$$ (6)

This quantity represents the expected number of secondary infections generated by a typical infectious individual in a fully susceptible population when transmission is reduced by non-pharmaceutical interventions through the factor (1−*c*_*n*_). Under this reference condition, no prior immunity or vaccine-induced protection is present, so the threshold depends only on the transmission rate β, the recovery rate γ, and the level of compliance with non-pharmaceutical interventions.

While $R_{0}$ characterizes transmission in a fully susceptible population, the relevant threshold for invasion must account for the population structure at the disease free state. In the present model, individuals may already be distributed across susceptible, waiting, and protected classes, which modifies the pool of individuals available for infection. This leads to the definition of the invasion reproduction number


$$\begin{array}{l} R_{\text{inv}}=\frac{\beta \left(1-c_{n}\right)}{\gamma }\frac{S_{\text{eff}}^{0}}{N_{0}}=R_{0}\frac{S_{\text{eff}}^{0}}{N_{0}}, \end{array}$$ (7)

where $S_{\text{eff}}^{0}$ in Equation 7 represents the effective susceptible population at the disease free state. This quantity accounts for heterogeneous susceptibility, with individuals in the protected class contributing through the factor ϵ, which reflects reduced susceptibility to infection.

Although $R_{\text{inv}}$ determines whether infection can invade near the disease free state, it does not capture how transmission evolves over time once an epidemic is underway. For this purpose, it is useful to introduce a time dependent reproduction number that reflects the current state of the population. This quantity, commonly referred to as the effective reproduction number. This measures the instantaneous transmission potential along epidemic trajectories and incorporates changes in susceptibility due to infection and vaccination.

Accordingly, we define the time dependent effective reproduction number as


$$\begin{array}{l} R_{e}\left(t\right)=\frac{\beta \left(1-c_{n}\right)}{\gamma }\frac{S_{a}\left(t\right)+W_{a}\left(t\right)+\epsilon P_{a}\left(t\right)+S_{h}\left(t\right)}{N\left(t\right)}. \end{array}$$ (8)

This expression given in Equation 8 generalizes the invasion threshold by replacing the disease free quantities with their time dependent counterparts, thereby capturing how transmission potential varies as the population is redistributed among epidemiological classes.

#### 2.6.3 Characteristic equation and local threshold

To determine the local stability of the disease free state, we analyze the linear delay in Equation 5 by seeking exponential solutions of the form


$$\begin{array}{l} I\left(t\right)=e^{\lambda t}, \end{array}$$ (9)

where λ ∈ ℂ. Substituting Equation 9 into Equation 5 gives


$$\begin{array}{l} \lambda e^{\lambda t}=\beta \left(1-c_{n}\right)\frac{S_{\text{eff}}^{0}}{N_{0}}e^{\lambda \left(t-\tau_{E}\right)}-\gamma e^{\lambda t}. \end{array}$$ (10)

Dividing through Equation 10 by *e*^λ*t*^ yields the characteristic equation


$$\begin{array}{l} \lambda +\gamma -\kappa e^{-\lambda \tau_{E}}=0, \end{array}$$ (11)

where


$$\kappa =\beta \left(1-c_{n}\right)\frac{S_{\text{eff}}^{0}}{N_{0}}.$$

The roots of Equation 11 determine the local behavior of solutions near the disease free state. In particular, the sign of the real part of λ establishes whether small perturbations growth (decay) over time. This leads to the following threshold result.

**Theorem 2** (Local threshold for the disease free state). *Let* $x^{0}\in D$ *be a disease free state. Then the following statements hold*.

*(i) If $R_{\text{inv}}<1$, then every root of Equation 11 satisfies ℜ(λ) < 0, and the disease free state is locally asymptotically stable.*

*(ii) If $R_{\text{inv}}>1$, then Equation 11 admits a positive real root, and the disease free state is unstable*.

*Proof:* Suppose that $R_{\text{inv}}<1$. Then κ < γ. Assume, for contradiction, that Equation 11 has a root λ such that ℜ(λ) ≥ 0. Taking absolute values in Equation 11 gives


$$|\lambda +\gamma |=\kappa e^{-ℜ\left(\lambda \right)\tau_{E}}\leq \kappa .$$

On the other hand,


|λ+γ|≥ℜ(λ+γ)=ℜ(λ)+γ≥γ.

Hence γ ≤ κ, which contradicts κ < γ. Therefore, every root satisfies ℜ(λ) < 0, and the disease free state is locally asymptotically stable.

Next, suppose that $R_{\text{inv}}>1$. Then κ>γ. Define


$$f\left(\lambda \right)=\lambda +\gamma -\kappa e^{-\lambda \tau_{E}},\;\lambda \in \mathbb{R}.$$

We have


f(0)=γ-κ<0,

and


$$\underset{\lambda \to \infty }{\lim}f\left(\lambda \right)=\infty .$$

By continuity, there exists λ^*^>0 such that *f*(λ^*^) = 0. Thus, Equation 11 has a positive real root, which implies instability of the disease free state.

This result shows that local stability is determined entirely by the sign of $R_{\text{inv}}-1$. We now examine how this threshold condition is reflected in the spectral structure of the characteristic equation through bifurcation analysis.

#### 2.6.4 Bifurcation analysis of the linearized infected subsystem

We examine how the effective transmission parameter and the incubation delay influence the spectral properties of the linearized infected subsystem. From the derivation in Equation 10, the total infectious population satisfies


$$\frac{dI}{dt}=\kappa I\left(t-\tau_{E}\right)-\gamma I\left(t\right),$$

with the associated characteristic equation given by


$$\begin{array}{l} \lambda +\gamma -\kappa e^{-\lambda \tau_{E}}=0. \end{array}$$ (12)

Thus, the stability of the disease free state is determined by the nature of the roots of Equation 12. The characteristic roots determine whether small perturbations growth (decay) over time. In particular, stability changes occur when characteristic roots cross the imaginary axis, while the sign of the dominant real part ℜ(λ) determines whether perturbations growth (decay) over time.

To examine how changes in transmission intensity affect the stability of the disease free state, we analyze the characteristic equation with respect to the effective transmission parameter κ. This parameter is used for the bifurcation analysis since it combines the transmission rate, the reduction due to non-pharmaceutical intervention compliance, and the effective susceptible population at the disease free state into the single invasion term of the linearized infected subsystem. Equivalently,


$$R_{\text{inv}}=\frac{\kappa }{\gamma },$$

so varying κ is equivalent to varying the invasion reproduction number when the recovery rate γ is fixed. This directly links the bifurcation analysis to epidemic threshold theory, where infection decays when $R_{\text{inv}}<1$ and epidemic growth occurs when $R_{\text{inv}}>1$. The following result then shows that the threshold case $R_{\text{inv}}=1$ is associated with a real eigenvalue crossing at the origin.

** Proposition 1** (Real root crossing at the threshold). *At the critical value*


$$\kappa =\gamma \;equivalently\;R_{\text{inv}}=1,$$

*Equation 12 admits the root λ = 0. This root is simple and crosses the origin transversally as κ varies through γ.*

*Proof:* Substituting λ = 0 into Equation 12 gives


γ-κ=0,

so λ = 0 is a root precisely when κ = γ. To verify simplicity, we define


$$F\left(\lambda ,\kappa \right)=\lambda +\gamma -\kappa e^{-\lambda \tau_{E}}.$$

Then


$$\frac{\partial F}{\partial \lambda }=1+\kappa \tau_{E}e^{-\lambda \tau_{E}}.$$

Evaluating at (λ, κ) = (0, γ) yields


$$\frac{\partial F}{\partial \lambda }\left(0,\gamma \right)=1+\gamma \tau_{E}>0,$$

which confirms that the root is simple. By the implicit function theorem, there exists a locally unique branch λ = λ(κ) such that λ(γ) = 0, and


$$\lambda^{\prime }(\gamma )=-\frac{\partial F/\partial \kappa }{\partial F/\partial \lambda }{|}_{\left(0,\gamma \right)}=\frac{1}{1+\gamma \tau_{E}}>0.$$

Thus, the real root crosses zero from negative to positive values as κ increases through γ.

** Proposition 2** (Absence of delay induced Hopf bifurcation in the stable regime). *If κ < γ, then Equation 12 admits no purely imaginary roots for any τ_*E*_ ≥ 0. Consequently, the disease free state does not lose stability through a Hopf bifurcation as τ_*E*_ varies in this regime*.

*Proof:* Suppose λ = *iω* with ω ∈ ℝ is a root of Equation 12. Substituting and separating real and imaginary parts gives


$$\gamma =\kappa \cos\left(\omega \tau_{E}\right),\;\omega =-\kappa \sin\left(\omega \tau_{E}\right).$$

Squaring and adding yields


$$\kappa^{2}=\gamma^{2}+\omega^{2}.$$

If κ < γ, then γ^2^+ω^2^ ≥ γ^2^>κ^2^, which is a contradiction. Hence no purely imaginary roots exist.

** Proposition 3** (Instability above threshold). *If κ>γ, then Equation 12 admits a positive real root for every τ_*E*_ ≥ 0. Therefore the linearized infected subsystem is unstable whenever* $R_{\text{inv}}>1$.

*Proof*: Define


$$f\left(\lambda \right)=\lambda +\gamma -\kappa e^{-\lambda \tau_{E}},\;\lambda \in \mathbb{R}.$$

If κ>γ, then


$$f\left(0\right)=\gamma -\kappa <0,\;\underset{\lambda \to +\infty }{\lim}f\left(\lambda \right)=+\infty .$$

By continuity, there exists λ^*^>0 such that *f*(λ^*^) = 0.

**Figure 2:**
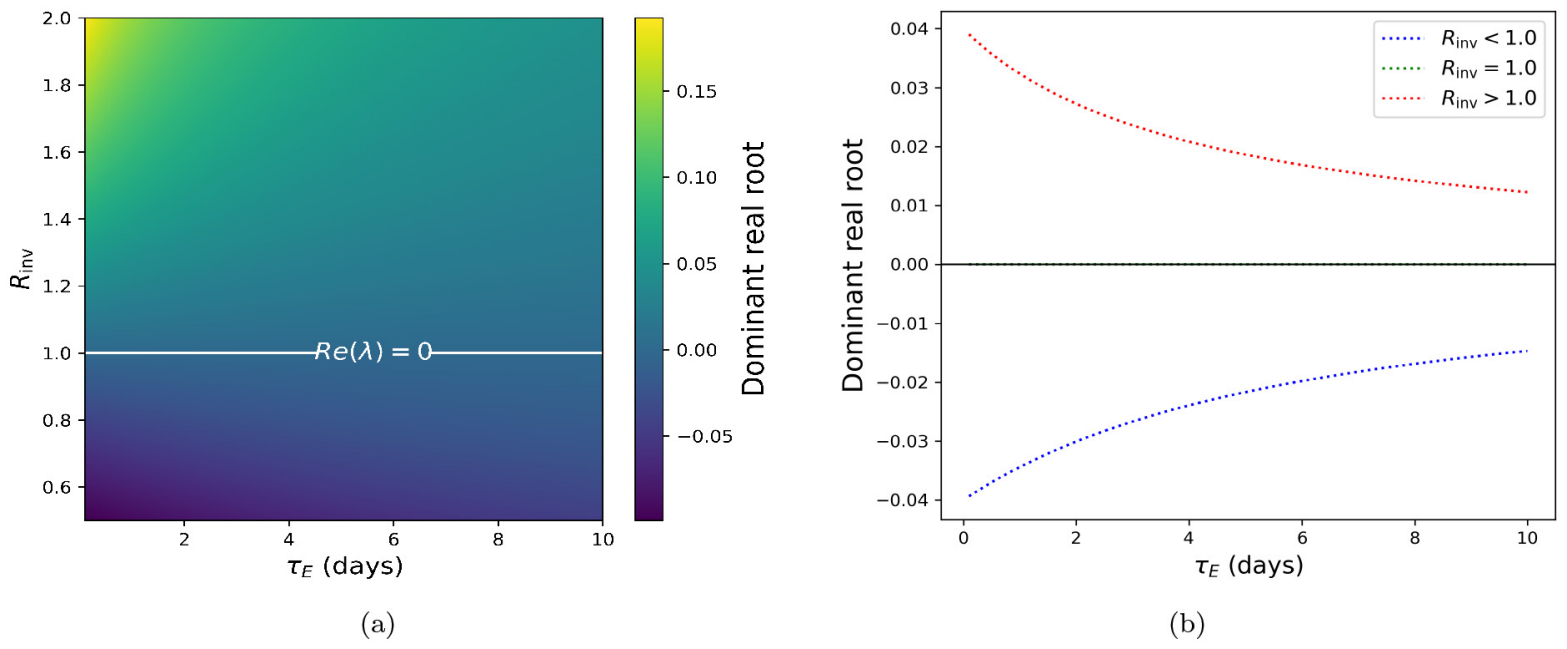
Bifurcation structure of the linearized infected subsystem. **(a)** Heat map of the dominant real part of the characteristic root in the $\left(\tau_{E},R_{\text{inv}}\right)$ plane. The contour ℜ(λ) = 0 identifies the stability boundary at $R_{\text{inv}}=1$. **(b)** Dominant real root as a function of τ_*E*_ for representative values of $R_{\text{inv}}$.

The above results show that the stability transition at $R_{\text{inv}}=1$ is generated by a real eigenvalue crossing zero. This establishes a transcritical bifurcation of the disease free equilibrium at the invasion threshold. The incubation delay τ_*E*_ enters the characteristic equation through an exponential term and therefore modifies the magnitude of the dominant root. As a result, it influences the rate of epidemic growth without altering the threshold condition. In addition, no delay induced Hopf bifurcation arises in the stable regime of the linearized infected subsystem. This is further shown in Figure 2.

Figure 2 illustrates the bifurcation structure. In Figure 2a, the zero contour of the dominant real part of the characteristic root occurs at $R_{\text{inv}}=1$ across the full range of τ_*E*_, which confirms that the stability boundary is independent of the incubation delay. Figure 2b further shows that increasing τ_*E*_ reduces the magnitude of the dominant root, which indicates slower decay when $R_{\text{inv}}<1$ and slower growth when $R_{\text{inv}}>1$. These observations are consistent with the analytical results and confirm that the bifurcation is governed by a real eigenvalue crossing.

Finally, the delays τ_*P*_ and τ_*V*_ do not appear in Equation 12, since they act only through the vaccination dynamics of uninfected compartments at the disease free state. Their influence therefore enters through the non-linear transient dynamics of the full system and is examined in the sensitivity analysis presented in the following section.

### 2.7 Sensitivity analysis

**Figure 3:**
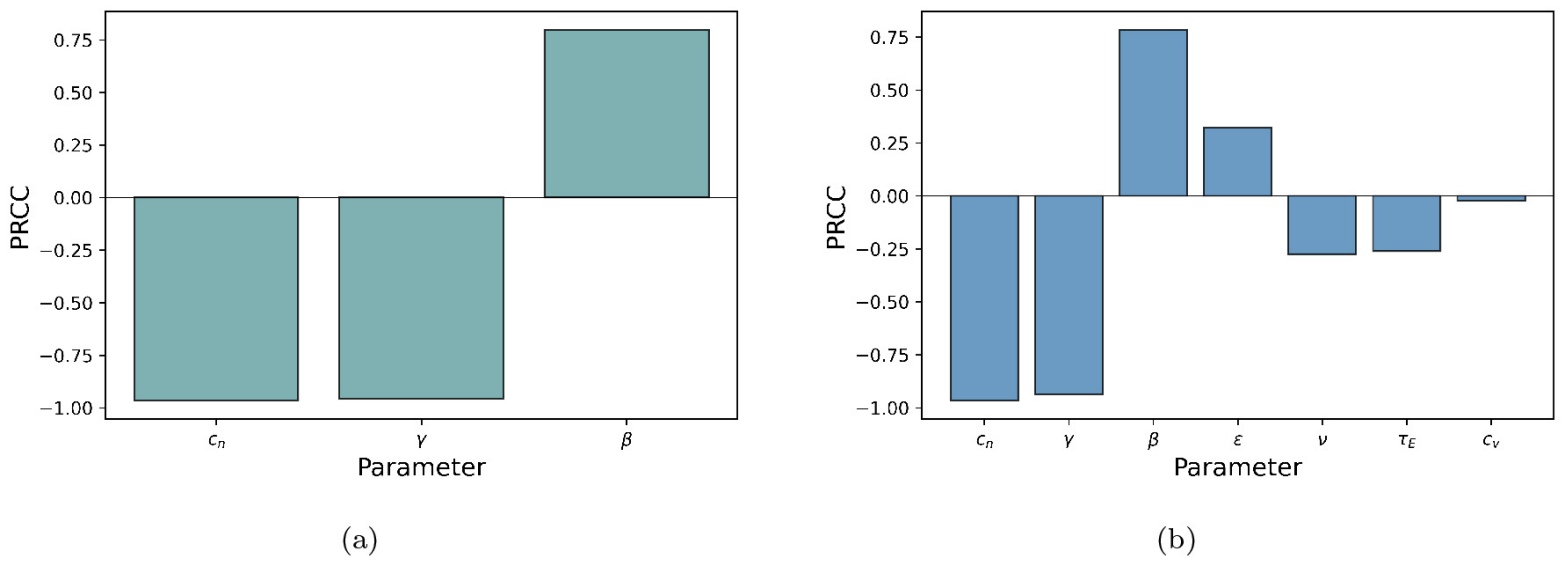
PRCC sensitivity of model parameters. **(a)** Partial rank correlation coefficients for the basic reproduction number. **(b)** Partial rank correlation coefficients for the cumulative infected proportion over 1 year. Positive values indicate parameters associated with increased outcome values, whereas negative values indicate parameters associated with reduced outcome values.

To quantify the influence of model parameters on transmission dynamics and epidemic dynamics, we conducted a global sensitivity analysis using Latin Hypercube Sampling with Partial Rank Correlation Coefficients. This approach enables systematic exploration of parameter uncertainty while accounting for non-linear relationships between model inputs and outputs. Two outcome measures were considered. The first is the basic reproduction number, which characterizes transmission potential under non-pharmaceutical intervention compliance. The second is the cumulative infected proportion over a 1 year period, obtained from numerical simulations of the full model under fixed pre-deployment and vaccine-to-protection delays. The results are summarized in Figure 3.

Figure 3a shows that $R_{0}$ is primarily driven by the transmission rate β, non-pharmaceutical intervention compliance *c*_*n*_, and the recovery rate γ. The positive PRCC associated with β indicates that increases in transmission intensity lead to higher values of $R_{0}$. In contrast, both *c*_*n*_ and γ exhibit strong negative associations, which reflects their role in reducing effective transmission and shortening the infectious period. This pattern is consistent with the analytical expression for $R_{0}$ in Equation 6, where these parameters directly determine the threshold.

The sensitivity of the cumulative infected proportion, shown in Figure 3b, follows a similar structure but reflects the full non-linear dynamics of the model. The dominant parameters remain β, *c*_*n*_, and γ, which indicates that epidemic burden is largely governed by transmission intensity and recovery processes. In addition, the residual susceptibility parameter ϵ has a positive association with cumulative infection, which shows that reduced vaccine effectiveness leads to increased infection burden.

Beyond these dominant effects, parameters associated with vaccination and delays contribute more modest but consistent influences. The vaccination rate ν exhibits a negative association with cumulative infection, which indicates that faster vaccine administration reduces outbreak size. The incubation delay τ_*E*_ also shows a negative association, which reflects the slowing of progression to infectiousness. In contrast, the vaccination compliance parameter *c*_*v*_ has a relatively small effect, which suggests that changes in uptake alone have limited impact when transmission intensity remains high.

These findings indicate that epidemic dynamics are most sensitive to parameters that directly regulate transmission and recovery, while vaccination related parameters influence outcomes through indirect mechanisms. This result provides a quantitative basis for prioritizing interventions that reduce transmission intensity, particularly during the early phase of an outbreak.

Having identified the parameters with the strongest influence on transmission dynamics, we next examine how variations in delay and intervention mechanisms shape epidemic dynamics through numerical simulations.

## 3 Results

This section presents numerical simulations of the time-delayed model to examine how delays in vaccination and disease progression, together with behavioral factors, influence outbreak size, peak timing, and group level infection burden. These simulations extend the analytical results previously developed by quantifying the effects of delays under finite time horizons. In particular, the analysis focuses on how biological delays interact with intervention dynamics to shape epidemic dynamics.

Parameter values were obtained from the literature and are summarized in Table 1. Specifically, the biological parameters were selected based on empirical estimates, while operational and behavioral parameters were specified using baseline values and plausible ranges to support scenario exploration. To ensure consistency between analytical and numerical results, the transmission rate β was computed from the basic reproduction number $R_{0}$ derived in Equation 6.

**Table 1:** Baseline parameter values and simulation ranges.

Parameter	Description	Baseline	Range used	Unit	Source
τ_*E*_	Incubation delay	2	1 - 5	Days	(55)
γ	Recovery rate	0.2	0.1 − 0.3	Day^−1^	(55, 56)
τ_*V*_	Vaccine-to-protection delay	14	7 − 35	Days	(57)
τ_*P*_	Pre-deployment delay	120	60 − 180	Days	(22, 23)
*R*_0_	Baseline reproduction number	1.7	1.3 − 2.0	Dimensionless	(56, 58)
β	Transmission rate	Computed	Implied by *R*_0_	Day^−1^	(59, 60)
*c*_*n*_	NPI compliance	0.40	0.00 − 0.70	Dimensionless	(22, 23)
ν	Vaccination rate	0.02	0.005 − 0.05	Day^−1^	(23)
*c*_*v*_	Vaccination compliance	0.70	0.50 − 0.90	Dimensionless	(23)
ϵ	Residual susceptibility	0.40	0.20 − 0.70	Dimensionless	(14, 61)

Delay parameters are measured in days, rate parameters are measured per day, while compliance, susceptibility, and reproduction numbers are dimensionless.

Using these parameter values, the model was solved numerically with constant delays and prescribed history functions on the interval [−τ, 0]. The simulations were designed to isolate the effects of delay parameters on epidemic dynamics while maintaining consistency with the theoretical formulation. For each parameter set, three outcome measures were computed. The peak infectious burden defined as $\underset{t\in \left[0,T\right]}{\max}I\left(t\right)$, the time to peak infection, and the cumulative epidemic burden measured by *R*(*T*). These metrics are used to provide complementary information on both the magnitude and timing of the outbreak, thereby enabling direct comparison across different delay and intervention scenarios. We begin by examining the effect of delays in vaccine availability on epidemic dynamics.

### 3.1 Effect of pre-deployment delay on outbreak size and peak timing

**Figure 4:**
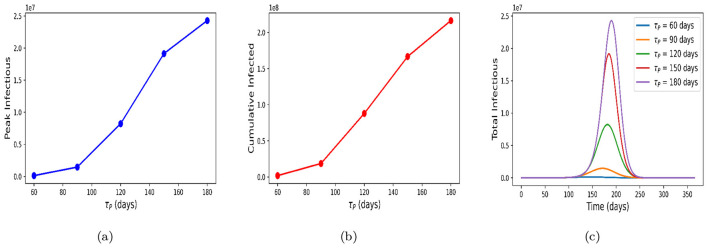
Effect of the pre-deployment delay τ_*P*_ on epidemic dynamics. **(a)** Peak infectious burden as a function of τ_*P*_. **(b)** Cumulative number of infected individuals over the simulation period. **(c)** Temporal evolution of the total infectious population for varying values of τ_*P*_.

We examine how the timing of vaccine availability influences epidemic dynamics by varying the pre-deployment delay τ_*P*_. This delay determines the period during which transmission proceeds without vaccine-induced protection. The impact of τ_*P*_ on peak infectious burden, cumulative infection, and temporal epidemic trajectories is shown in Figure 4.

Figure 4a shows that the peak infectious burden increases monotonically with τ_*P*_. This pattern arises because early vaccination reduces the number of susceptible individuals available for infection during the initial growth phase. When τ_*P*_ is small, vaccination begins before transmission accelerates, which limits the buildup of infectious individuals. As τ_*P*_ increases, vaccination is delayed and transmission proceeds with minimal intervention, leading to a larger infectious population and a higher epidemic peak. The cumulative number of infections exhibits a similar dependence on τ_*P*_, as shown in Figure 4b. Larger delays result in greater cumulative burden, which reflects the accumulation of infections before vaccination begins. When vaccination is introduced late, a substantial fraction of the population has already been infected, which reduces the capacity of vaccination to prevent additional cases.

The temporal dynamics in Figure 4c provide further clarification of this mechanism. Increasing τ_*P*_ leads to steeper epidemic curves and earlier attainment of higher peak levels. In contrast, smaller values of τ_*P*_ produce slower epidemic growth and lower peaks, since vaccination begins early enough to alter transmission dynamics before widespread infection occurs. These results demonstrate that the timing of vaccine deployment is a primary determinant of both outbreak magnitude and timing. Early deployment limits transmission during the initial growth phase and reduces both peak burden and cumulative infections. Delayed deployment allows the epidemic to progress largely without intervention, which results in larger and more rapidly growing outbreaks.

While the pre-deployment delay governs when vaccination begins, disease progression itself is also subject to intrinsic biological delays. We therefore next examine how the incubation delay influences outbreak size and peak timing.

### 3.2 Effect of incubation delay on outbreak size and peak timing

**Figure 5:**
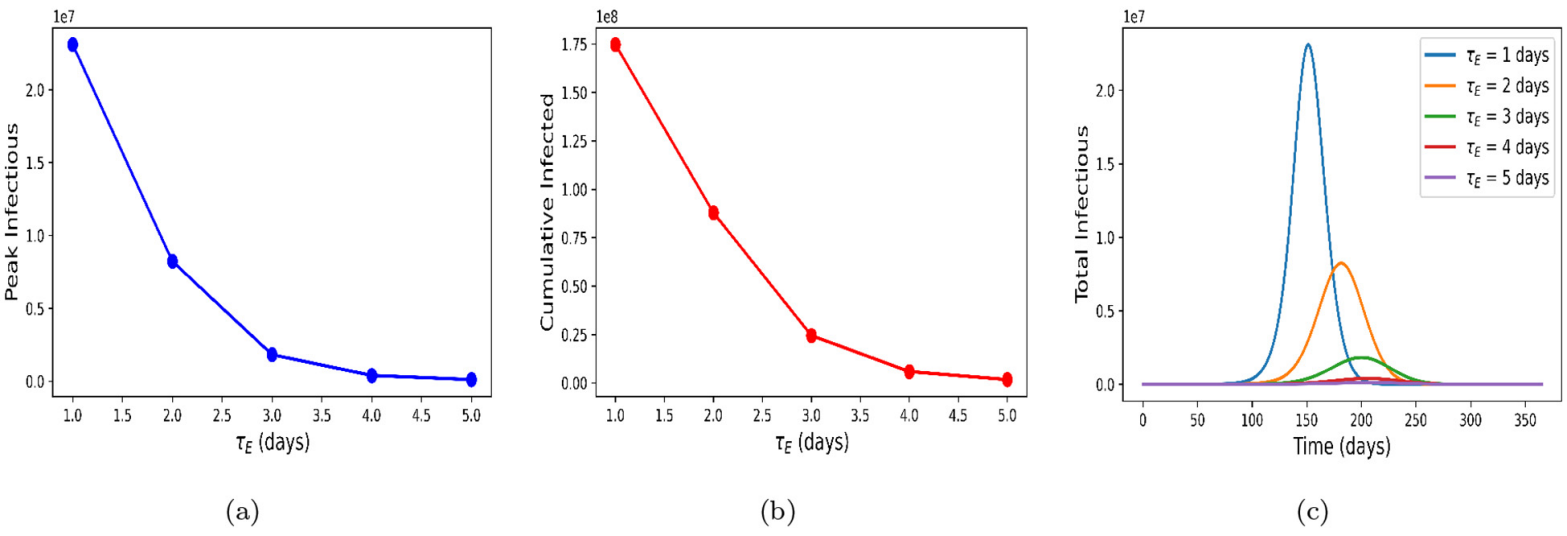
Influence of the incubation delay τ_*E*_ on epidemic dynamics. **(a)** Peak infectious burden as τ_*E*_ varies. **(b)** Cumulative number of infected individuals over the simulation period. **(c)** Temporal evolution of the total infectious population for varying values of τ_*E*_.

We examine how the incubation delay τ_*E*_ influences outbreak size and peak timing. This delay governs the time between infection and the onset of infectiousness, and influence the rate at which new infections contribute to transmission. The impact of τ_*E*_ on peak infectious burden, cumulative infections, and temporal epidemic trajectories is shown in Figure 5.

Figure 5a shows an inverse relationship between τ_*E*_ and the peak infectious burden. A shorter incubation delay allows individuals to become infectious more rapidly, which accelerates transmission during the early phase of the epidemic and leads to a higher peak. In contrast, increasing τ_*E*_ delays the transition to infectiousness, which slows the rate at which new infections are generated and results in a lower maximum infectious level. This effect extends to the cumulative number of infections, as shown in Figure 5b. Large τ_*E*_ leads to a reduction in cumulative burden. This reduction partly occurs because delayed infectiousness distributes transmission over a longer time interval, which decreases the intensity of transmission at any given time and limits the total number of infections.

The temporal trajectories in Figure 5c further illustrate this mechanism. Increasing τ_*E*_ shifts the epidemic peak to later times and produces broader infection curves, which indicates that transmission is sustained over a longer duration. In contrast, smaller values of τ_*E*_ produce sharper and earlier peaks, reflecting more rapid epidemic growth. These numerical findings are consistent with the analytical results, where the incubation delay appears in the infected subsystem through the delayed infection term. This delay affects the timing of secondary transmission but does not alter the basic reproduction number. Consequently, τ_*E*_ modifies transient epidemic behavior while leaving the invasion threshold unchanged, thereby influencing both the timing and magnitude of outbreak dynamics.

While the incubation delay governs the progression from infection to infectiousness, vaccination introduces an additional delay before protection becomes effective. Next, we consider the effect of the vaccine-to-protection delay on outbreak size and peak timing.

### 3.3 Effect of vaccine-to-protection delay on outbreak size and peak timing

**Figure 6:**
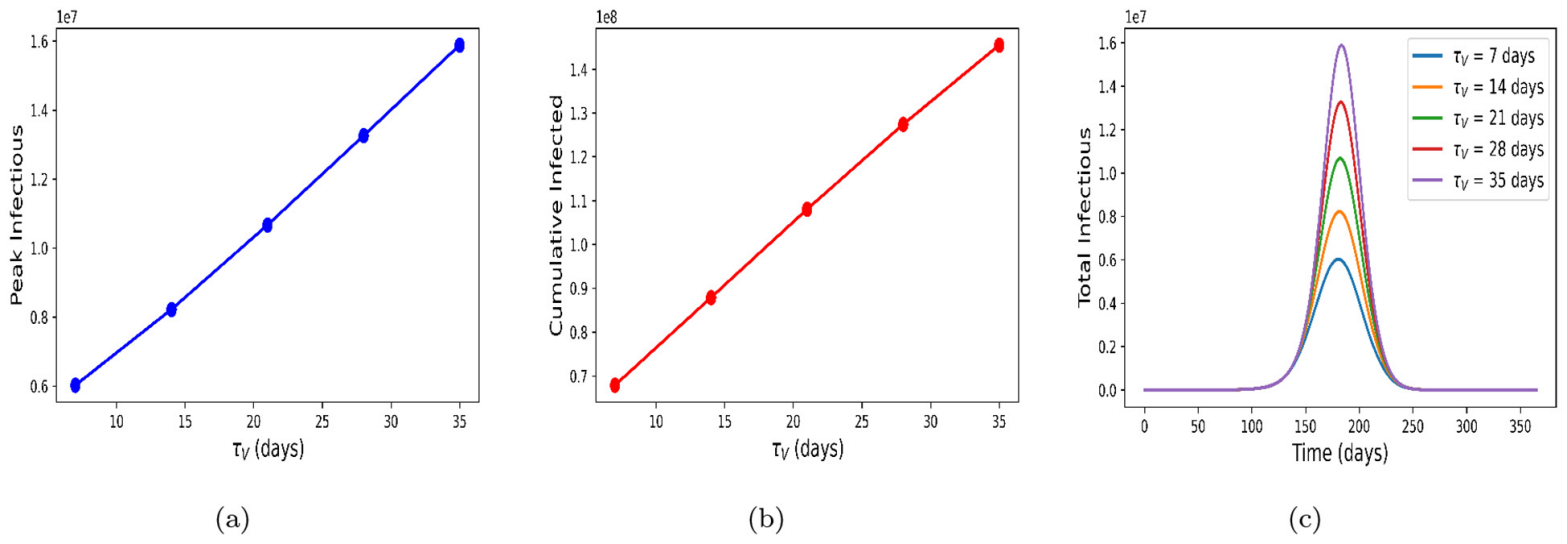
Effect of the vaccine-to-protection delay τ_*V*_ on epidemic dynamics. **(a)** Peak infectious burden as a function of τ_*V*_. **(b)** Cumulative number of infected individuals over the simulation period. **(c)** Temporal evolution of the total infectious population for varying values of τ_*V*_.

We assess how the vaccine-to-protection delay τ_*V*_ influences outbreak size and peak timing. This delay represents the time required for vaccinated individuals to develop effective immunity, which determines how quickly vaccination translates into reduced susceptibility. The impact of τ_*V*_ on peak infectious burden, cumulative infections, and temporal epidemic trajectories is shown in Figure 6.

Figure 6a shows that the peak infectious burden increases monotonically with τ_*V*_. This pattern arises from the delayed development of vaccine-induced protection. Since individuals in the waiting class remain fully susceptible until protection becomes effective. When τ_*V*_ is large, vaccinated individuals continue to contribute to transmission during the waiting period, thereby allowing infections to accumulate and producing a higher epidemic peak. The cumulative number of infections exhibits a comparable trend as shown in Figure 6b. Large τ_*V*_ leads to increased cumulative burden, which reflects the extended interval during which vaccinated individuals do not yet benefit from protection. As a result, ongoing exposure during this period generates additional infections and reduces the ability of vaccination to effectively prevent cases. The temporal trajectories in Figure 6c provide further clarification of this mechanism. Increasing τ_*V*_ raises the height of the epidemic peak while leaving the overall timing of the outbreak within a similar range. This pattern indicates that delayed immune protection primarily amplifies transmission intensity rather than substantially shifting the timing of the epidemic.

These results demonstrate that the effectiveness of vaccination depends not only on coverage but also on the speed at which immunity develops. When the delay to protection is large, vaccinated individuals remain susceptible for longer periods and contribute to transmission, which limits the short term impact of vaccination. Consequently, larger values of τ_*V*_ lead to higher peak infection levels and greater cumulative burden. Since the pre-deployment delay determines when vaccination begins and the vaccine-to-protection delay determines when immunity becomes effective, their combined influence is expected to shape epidemic dynamics in a coupled manner. We therefore next examine the joint effects of these delays.

### 3.4 Combined effects of pre-deployment and protection delays

**Figure 7:**
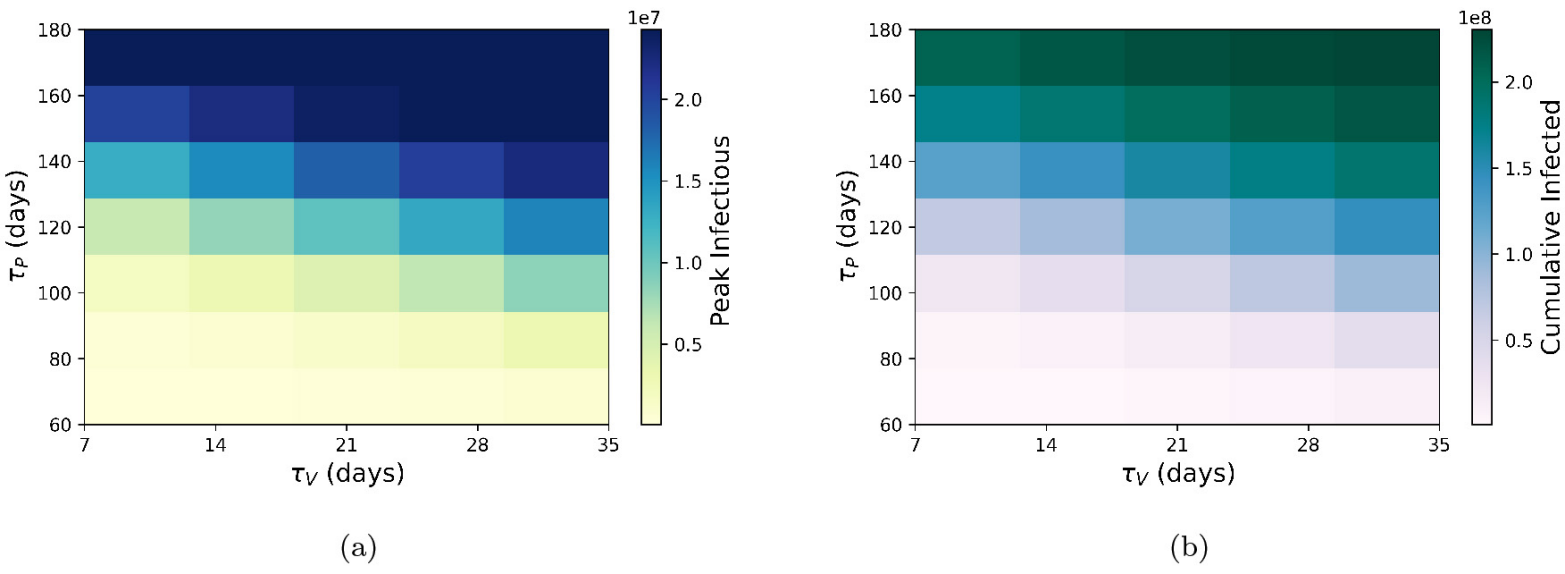
Joint influence of the pre-deployment delay τ_*P*_ and vaccine-to-protection delay τ_*V*_ on epidemic dynamics. **(a)** Peak infectious burden across the two-delay parameter space. **(b)** Cumulative number of infected individuals over the simulation period. The heat maps show how rollout timing and immune protection delay jointly shape peak intensity and total epidemic size.

We examine the joint influence of the pre-deployment delay τ_*P*_ and the vaccine-to-protection delay τ_*V*_ on epidemic dynamics. These delays act at different stages of the vaccination process, with τ_*P*_ determining when vaccination begins and τ_*V*_ determining when protection becomes effective. Their combined effect is therefore expected to shape both the timing and magnitude of epidemic dynamics. The resulting patterns are shown in Figure 7, where heat maps display variations in peak infectious burden and cumulative infection across the two dimensional delay space.

Figure 7a shows that the peak infectious burden increases as either τ_*P*_ or τ_*V*_ increases. The lowest peak values occur when vaccination is initiated early and protection develops rapidly. This pattern reflects the complementary roles of the two delays. The pre-deployment delay controls whether vaccination is able to influence early transmission, while the vaccine-to-protection delay determines how quickly vaccinated individuals reduce their susceptibility after rollout begins. The cumulative number of infections exhibits a consistent pattern, as shown in Figure 7b. The smallest epidemic sizes occur in regions where both delays are minimal, whereas larger values of τ_*P*_ and τ_*V*_ correspond to increased cumulative burden. This increase reflects the reduced ability of vaccination to prevent infections when transmission has already progressed or when protection is not achieved in a timely manner.

The interaction between the two delays is not uniform across the parameter space. When the pre-deployment delay is large, increasing τ_*V*_ produces only modest additional changes, since a substantial fraction of transmission has already occurred before vaccination begins. In contrast, when vaccination is initiated early, variations in τ_*V*_ have a stronger effect on both peak burden and cumulative infection. This indicates that the timing of rollout determines the extent to which delays in immune protection influence epidemic dynamics.

These findings demonstrate that vaccination effectiveness depends jointly on deployment timing and the rate of immune protection. Early rollout combined with rapid protection yields the greatest reduction in both peak infection and cumulative burden. In contrast, concurrent delays in these processes substantially weaken the impact of vaccination and lead to larger outbreaks. While the previous simulations focuses on delays in implementation and protection, population-level outcomes are also shaped by behavioral factors such as vaccination acceptance. We therefore next examine how pre-deployment delay interacts with vaccination compliance.

### 3.5 Interaction between pre-deployment delay and vaccination acceptance

**Figure 8:**
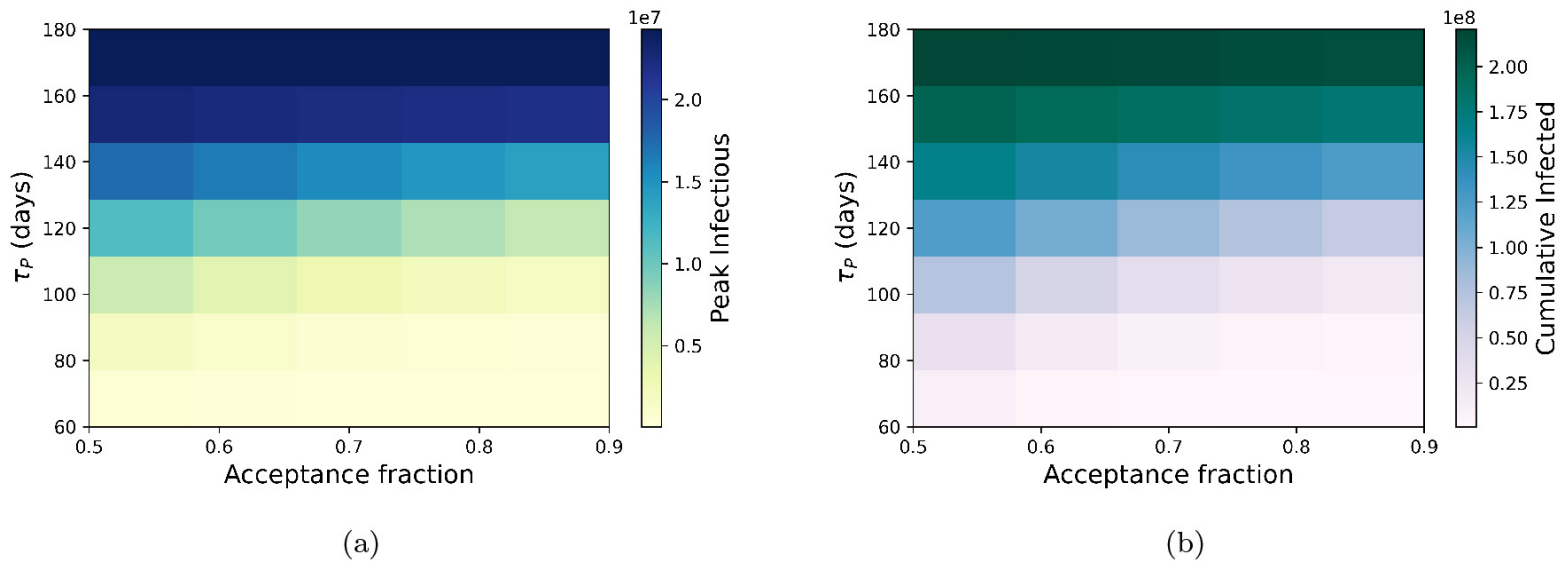
Dependence of epidemic dynamics on vaccination acceptance and pre-deployment delay τ_*P*_. **(a)** Peak infectious burden across combinations of acceptance levels and deployment delays. **(b)** Cumulative number of infected individuals over the simulation time horizon.

We investigate how the pre-deployment delay τ_*P*_ interacts with vaccination acceptance to influence epidemic dynamics. While τ_*P*_ determines when vaccination begins, the acceptance level controls the fraction of the population that enters the vaccination pathway. Their combined effect therefore determines the extent to which vaccination can reduce transmission. The resulting variation in peak infectious burden and cumulative infection is shown in Figure 8.

Figure 8a shows that increasing vaccination acceptance reduces the peak infectious burden when vaccination is implemented early. This reduction occurs because a larger fraction of susceptible individuals transitions into the vaccinated pathway, which decreases the effective susceptible population during the initial growth phase and limits transmission. As the pre-deployment delay increases, this relationship weakens. For sufficiently large delays, such as τ_*P*_ ≥ 120 days, the peak infectious burden varies only slightly across different acceptance levels. This pattern indicates that a substantial portion of transmission has already occurred before vaccination begins, which limits the ability of increased uptake to alter the epidemic trajectory.

The cumulative number of infections exhibits a consistent pattern, as shown in Figure 8b. When vaccination is introduced early, higher acceptance leads to a substantial reduction in total infections. As τ_*P*_ increases, the cumulative burden approaches levels associated with minimal intervention impact, and differences across acceptance levels become less pronounced. These results demonstrate that vaccination acceptance and deployment timing act jointly in determining epidemic dynamics. High acceptance produces meaningful reductions in both peak burden and cumulative infection only when vaccination begins early enough to influence the initial phase of transmission. When deployment is delayed, the benefit of increased acceptance is substantially reduced because early infections have already occurred.

Next, we consider the temporal dynamics of transmission and the distribution of infection burden across behavioral groups. This is achieved through analysis of the effective reproduction number and group specific infection patterns.

### 3.6 Effective reproduction number and group level infection burden

**Figure 9:**
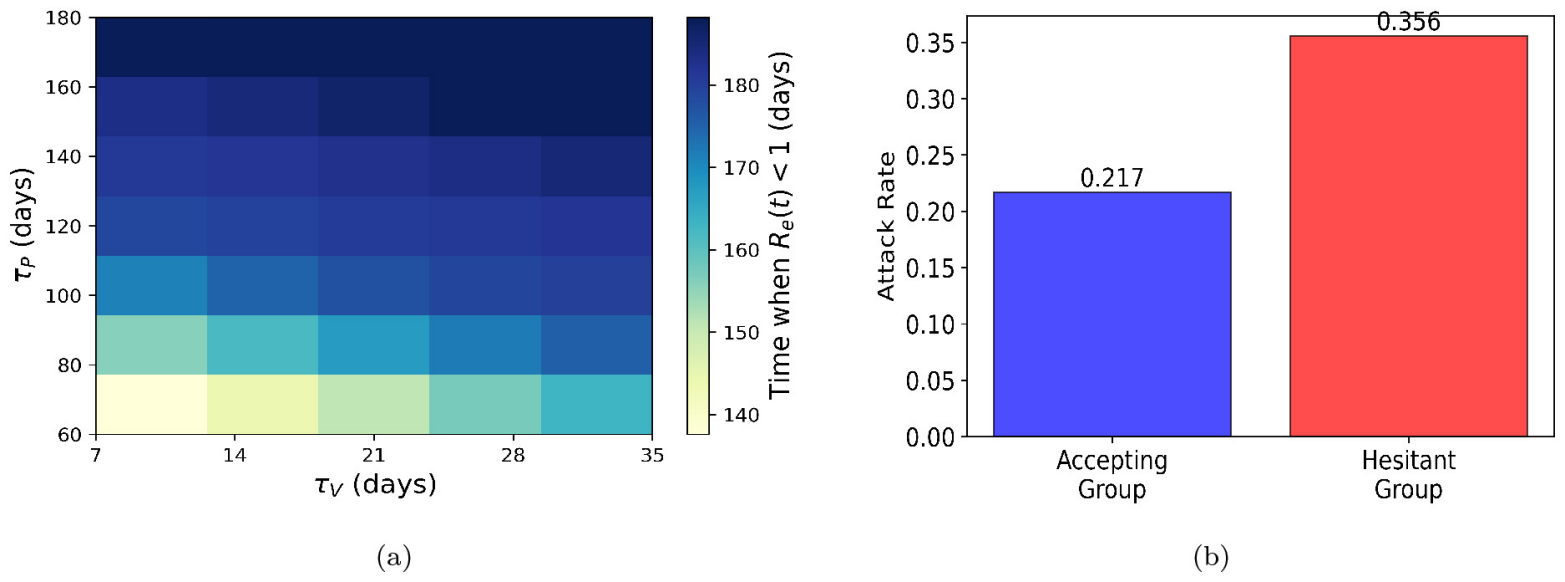
Relationship between the effective reproduction number and group-level infection burden. **(a)** Regions where the effective reproduction number *R*_*e*_(*t*) falls below one over time for different pre-deployment delays. **(b)** Attack rates for the vaccine accepting and vaccine hesitant groups over the simulation horizon.

We examine the relationship between transmission dynamics and infection burden through threshold behavior and group specific outcomes. In particular, we analyze the time dependent effective reproduction number together with measures of infection burden across population groups. The corresponding results are presented in Figure 9.

Figure 9a identifies regions in the (*t*, τ_*P*_) plane where the effective reproduction number *R*_*e*_(*t*) falls below one. For smaller values of the pre-deployment delay, this threshold is reached earlier, which indicates that vaccination begins sufficiently early to reduce the effective susceptible population during the initial growth phase. As a result, transmission declines before a large infectious burden develops. As the pre-deployment delay increases, the timing of this threshold shifts to later periods. Under these conditions, the epidemic progresses with limited intervention during its early phase, and the reduction in *R*_*e*_(*t*) occurs only after a substantial fraction of the population has already been infected. This delayed crossing reflects both the late onset of vaccination and the depletion of susceptible individuals in the population.

To assess how these transmission dynamics translate into epidemiological outcomes, Figure 9b quantifies the cumulative infection burden in each group through the attack rate. The attack rate is approximately 0.217 in the vaccine-accepting group and 0.356 in the vaccine-hesitant group over the simulation horizon. This difference indicates that individuals in the hesitant group experience a higher probability of infection despite being subject to the same force of infection. These results show that delayed reduction in transmission leads to unequal infection burden across behavioral groups. Early suppression of *R*_*e*_(*t*) limits infections in both groups, whereas delayed control disproportionately increases infection risk among individuals who do not benefit from vaccination.

Next, we analyze how non-pharmaceutical interventions and vaccine breakthrough alter transmission dynamics and epidemic dynamics.

### 3.7 Effects of non-pharmaceutical interventions and vaccine residual susceptibility

**Figure 10:**
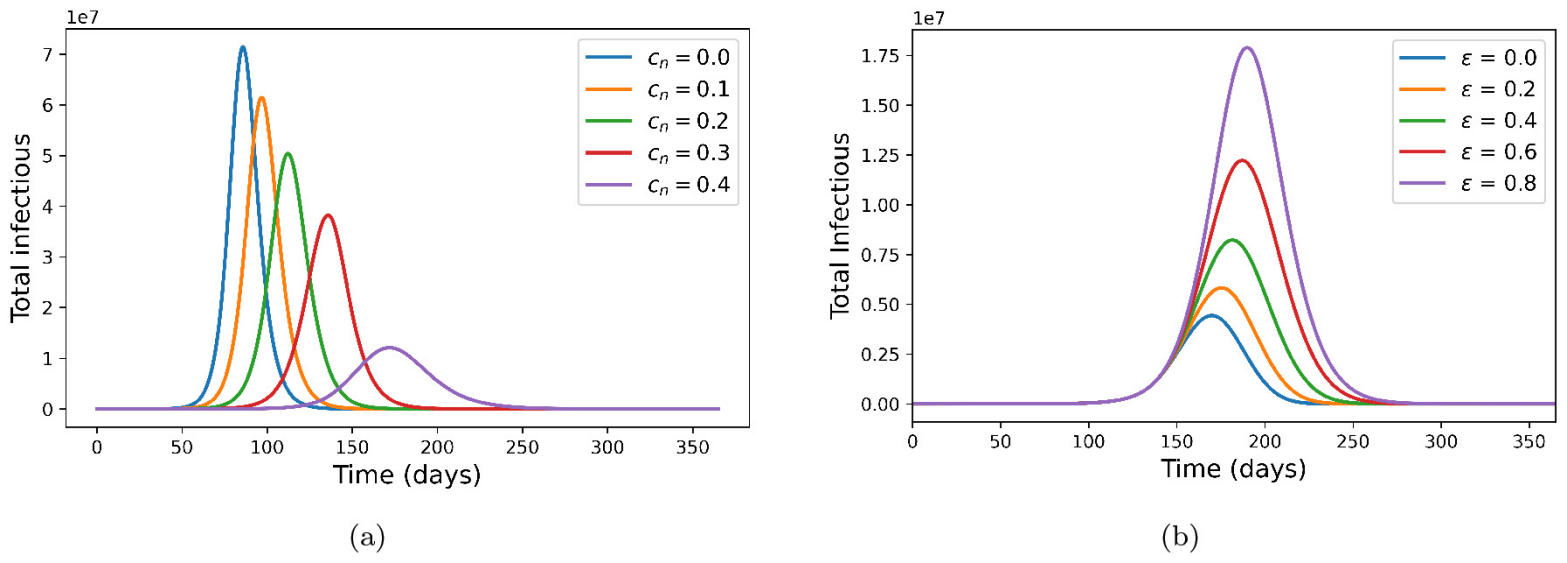
Effects of non-pharmaceutical compliance and vaccine residual susceptibility on epidemic dynamics. **(a)** Total infectious population for varying compliance levels *c*_*n*_. **(b)** Total infectious population for varying residual susceptibility ϵ, where larger ϵ corresponds to weaker vaccine-induced protection.

We examine how non-pharmaceutical intervention compliance and vaccine residual susceptibility influence epidemic dynamics. These factors operate through distinct mechanisms, with compliance reducing transmission at the population-level and breakthrough susceptibility affecting protection at the individual-level. The resulting temporal profiles of the infectious population are shown in Figure 10.

Figure 10a shows that increasing non-pharmaceutical compliance leads to a reduction in the infectious burden over time. This effect arises from a decrease in the effective transmission rate, which lowers the rate at which new infections are generated. Consequently, the epidemic peak is reduced and the growth of infection slows. The peak also occurs at later times, which indicates that transmission is progressively suppressed as compliance increases. The effect of vaccine breakthrough is shown in Figure 10b. As the residual susceptibility ϵ increases, the infectious burden rises across the time horizon. This pattern reflects reduced vaccine effectiveness, since individuals in the protected class retain a higher probability of infection. As a result, vaccinated individuals contribute more to ongoing transmission when ϵ is large.

These mechanisms influence epidemic dynamics in complementary ways. Increasing *c*_*n*_ reduces exposure risk across the population and lowers transmission intensity, whereas increasing ϵ weakens protection and raises susceptibility among vaccinated individuals. The combined effect determines the overall epidemic burden, with higher compliance and lower residual susceptibility producing the greatest reduction in infection levels.

## 4 Discussion

This study examined how explicit time delays, vaccination behavior, and non-pharmaceutical interventions jointly shape HPAI outbreak dynamics within a structured epidemic model. The results show that epidemic dynamics are governed not only on biological parameters but also on the timing of interventions and population behavior. In particular, delays in vaccination reduce its effectiveness during the early phase of an epidemic, as a substantial portion of transmission occurs before immune protection is established. Consequently, vaccination contributes less to limiting peak burden and instead plays a larger role in reducing infections during later stages. This pattern is consistent with previous studies showing that delayed interventions primarily affect transmission after substantial epidemic growth has occurred (22, 50, 51).

Behavioral heterogeneity further modifies these dynamics. By distinguishing between vaccine-accepting and hesitant individuals, the model shows that infection risk remains higher in the hesitant group across all scenarios which occur because hesitant individual does not benefit from vaccine-induced protection and therefore maintain a larger effective susceptible pool throughout the epidemic. As a result, infections continue to accumulate more rapidly in this group. The magnitude of this difference depends on vaccine performance, as higher residual susceptibility reduces the overall protective effect and narrows the gap between groups. These findings highlight that behavioral uptake and vaccine efficacy jointly determine both total burden and its distribution across the population.

Non-pharmaceutical interventions influence transmission though a distinct and complementary mechanism. Increased compliance reduces transmission immediately by lowering the effective contact rates, thereby limiting new infections at the outset of an outbreak. This contrasts with vaccination, which requires time to generate protection. As a result, non-pharmaceutical interventions exert a stronger influence during the early growth phase of the epidemic. Similar patterns were observed during the COVID-19 pandemic, where early reductions in transmission were driven by behavioral measures prior to widespread vaccine availability (52–54). This comparison reinforces the role of behavioral interventions in early outbreak control.

The interaction between intervention timing and behavioral response further clarifies how different strategies shape epidemic dynamics. Early vaccine deployment reduces transmission before substantial epidemic growth, allowing even moderate acceptance levels to produce meaningful reductions in burden. In contrast, delayed deployment limits the impact of increased acceptance, as early transmission has already occurred. Although non-pharmaceutical interventions can partially compensate for this limitation, their effectiveness depends on sustained compliance. This result demonstrate that distinct intervention combinations can yield similar cumulative outcomes while producing markedly different peak burdens and epidemic timing.

Biological delays also influence the temporal structure of the epidemic. Increasing the incubation delay spreads transmission over a longer period, reducing peak intensity, whereas increasing the vaccine-to-protection delay prolongs susceptibility among vaccinated individuals and increases peak burden. Although these delays do not alter the invasion threshold, they substantially affect transient epidemic dynamics, emphasizing the importance of explicitly representing delay processes in epidemic models.

It is important to note that this analysis focuses on a single epidemic wave without demographic turnover, reflecting the relatively rapid progression of HPAI outbreaks in humans. Within this time frame, infection and recovery processes operate on shorter time scales than demographic changes such as births and natural deaths. Under these scenarios, the results show that vaccination timing, immune delays, and behavioral compliance jointly determine both the magnitude and timing of epidemic dynamics.

## 5 Conclusion

This study developed a time-delayed epidemic model to examine how vaccination timing, behavioral responses, and non-pharmaceutical interventions influence the control of HPAI outbreaks. By incorporating delays in vaccine deployment, immune protection, and disease progression together with heterogeneity in vaccination acceptance, the model captures key mechanisms shaping epidemic dynamics. The results show that delays in vaccination substantially reduce the ability of vaccines to limit peak infection burden, even when efficacy is high. Early deployment reduces transmission during the initial growth phase, whereas delayed rollout shifts the impact of vaccination to later stages of the epidemic. Vaccination acceptance further influences both total infection burden and the distribution of risk, with lower acceptance associated with higher attack rates.

Non-pharmaceutical interventions provide an immediate reduction in transmission and therefore play a critical role during early phase of an outbreak. Higher compliance reduces peak burden and slows epidemic growth, which complements the delayed effect of vaccination. However, this benefit depends on sustained behavioral adherence, particularly when vaccination is delayed or immune protection develops slowly. Overall, these findings demonstrate that effective epidemic control depends on the interaction between intervention timing, immune response delays, and population behavior. Incorporating these factors into epidemic models provides a more realistic basis for evaluating control strategies and improving public health preparedness.

## 6 Future work

While the present study focuses on short-term epidemic dynamics, this choice reflects the rapid progression of HPAI outbreaks in humans, where transmission and recovery occur on a much shorter time scale than demographic processes such as births and natural deaths. For this reason, the model emphasizes processes that govern a single epidemic wave. Several extensions can improve the applicability of the model and broaden its use in epidemic analysis. In particular, the current formulation assumes a closed population without demographic turnover. Extending the model to include births, natural deaths, and waning immunity would allow the investigation of longer time scale dynamics. However, for HPAI H5N1 in humans, sustained endemic transmission has not been observed, so such an extension should be interpreted cautiously and regarded mainly as a methodological generalization rather than a direct representation of current epidemiological patterns.

The model also assumes homogeneous mixing within each subpopulation. Relaxing this assumption through age-structured mixing or network-based interactions would provide a more realistic representation of transmission pathways and improve interpretation of group-specific infection dynamics. Similarly, vaccine effects are currently represented by a fixed residual susceptibility parameter. Extending this to include time-dependent immunity, dose effects, or waning protection would allow more detailed analysis of vaccination strategies, including booster schedules and temporal variation in vaccine performance. Finally, the current model is deterministic and does not account for stochastic effects stochastic effects, which can be important during the early stages of an outbreak when case numbers are low. Incorporating stochasticity would allow quantification of variability in epidemic trajectories and estimation of probability of outbreak extinction under comparable conditions. Such an extension would improve the model's relevance for real-world outbreak risk assessment and decision-making under uncertainty.

## Data Availability

Data sharing is not applicable to this article as no datasets were generated or analyzed for the study. Code Availability: The code used to generate the simulations and plots in this study is openly available in the GitHub repository at https://github.com/Oluwatosin-babasola/DELAYED_HPAI.
